# Seasonal variations and the COVID-19 pandemic: impact on antimicrobial stewardship and antibiotic prescribing in a UK secondary care setting to combat antimicrobial resistance—a pilot study

**DOI:** 10.3389/fmicb.2025.1530414

**Published:** 2025-03-28

**Authors:** Rasha Abdelsalam-Elshenawy, Nkiruka Umaru, Zoe Aslanpour

**Affiliations:** School of Health, Medicine and Life Sciences, University of Hertfordshire, Hatfield, United Kingdom

**Keywords:** antimicrobial stewardship (AMS), antimicrobial resistance, COVID-19 pandemic, seasonal variation, hospital, antibiotic stewardship (ABS), antimicrobial stewardship (ASP) intervention, antibiotic prescribing

## Abstract

Antimicrobial resistance (AMR) remains a significant global health challenge, exacerbated by inappropriate antibiotic use, particularly during crises such as the COVID-19 pandemic. This pilot study evaluates the impact of seasonal variations and the pandemic on antimicrobial stewardship (AMS) practices in a UK secondary care setting. Using an interrupted time-series analysis, the study examined antibiotic prescribing patterns for respiratory tract infections (RTIs) during the pre-pandemic period of 2019 and the pandemic year of 2020. Among the 80 admissions reviewed, communityacquired pneumonia (CAP) was the most frequent diagnosis, with cases peaking at 15 in December 2019, illustrating the seasonal burden of RTIs. AMS interventions were assessed using the CARES framework, as recommended by the United Kingdom Health Security Agency’s (UKHSA) Start Smart, Then Focus toolkit. This CARES framework consists of five key actions: Cease, which involves discontinuing antibiotics if no infection is present; Amend, modifying antibiotic therapy based on clinical response or diagnostic findings; Refer, consulting specialised services when additional expertise is required; Extend, continuing antibiotic therapy with a documented review date; and Switch, transitioning from intravenous to oral antibiotic therapy when clinically appropriate. Notable shifts in AMS practices were observed, with Cease interventions increasing from 5% in winter 2019 to 9% by early spring 2020, Amend actions briefly spiking in March 2020, and Switch interventions peaking at 6% in spring 2020, reflecting dynamic stewardship responses to the evolving pandemic landscape. While the small sample size limits statistical power, a more extensive validation sample would strengthen the robustness of the data extraction tool and enhance its credibility for broader applications. Nevertheless, these findings highlight the importance of adaptive, sustainable, and resilient AMS strategies that align with seasonal trends to mitigate AMR risks and ensure effective healthcare delivery during public health emergencies. The study highlights the value of pilot testing in ensuring feasibility and reliability, advocating for the development of robust AMS frameworks to combat AMR and build healthcare resilience during future global crises.

## Introduction

Antimicrobial resistance (AMR) is a critical global health threat, causing an estimated 4.71 million deaths in 2021, with mortality trends varying significantly by age and region. Although AMR deaths among children under five have decreased, rates among older adults have sharply increased, driven largely by multidrug-resistant bacteria such as methicillin-resistant *Staphylococcus aureus* and carbapenem-resistant Gram-negative pathogens (Kariuki, 2024). Globally, multidrug-resistant *Acinetobacter baumannii* has also emerged, especially in Brazil, associated with invasive medical procedures, antibiotic use, and severe infections (Silva et al., 2022). AMR threatens progress towards the United Nations Sustainable Development Goals (SDGs), particularly SDG 3, which aims to ensure healthy lives and promote well-being for all (Jasovský et al., 2016).

This hidden crisis necessitates immediate and sustained action to prevent a future where common infections become untreatable and medical procedures carry increased risks (World Health Organization, 2023). The introduction of penicillin in the 1920s marked a transformative era in infection management, significantly reducing mortality rates (Elshenawy et al., 2023a,b). However, despite these advancements, inappropriate antibiotic prescriptions have driven the rise of AMR (GOV.UK, 2024). Urgent and sustainable measures are essential to combat AMR and preserve the effectiveness of antibiotics (GOV.UK, 2024).

Seasonal variations significantly impact antibiotic prescribing patterns, often resulting in higher rates of inappropriate antibiotic use, particularly for conditions where antibiotics are rarely indicated. Antibiotic prescribing increases notably during winter, frequently without clear clinical justification, thereby exacerbating the AMR crisis. Recognising these seasonal prescribing patterns provides opportunities for targeted AMS interventions aimed at reducing inappropriate antibiotic use and strengthening stewardship efforts during critical periods (Serletti et al., 2023).

Antimicrobial stewardship (AMS) is a pivotal component of the UK’s five-year strategy to effectively combat antimicrobial resistance. Its implementation promotes judicious antibiotic use, optimises treatment outcomes, and minimises resistance (Elshenawy et al., 2023a,b). In 2015, Public Health England (PHE) developed the “Start Smart, Then Focus” (SSTF) toolkit, a structured, evidence-based approach guiding AMS practices in inpatient care. The toolkit provides guidance for clinicians and healthcare leaders to reduce AMR risks while maintaining high-quality patient care. In 2023, the UK Health Security Agency (UKHSA) updated the SSTF toolkit, emphasising timely and responsible antibiotic use through the rapid initiation of effective therapies. The SSTF approach consists of two key phases: Start Smart, which focuses on the prompt and appropriate initiation of antibiotic therapy, and Then Focus, which involves reviewing and adjusting therapy based on clinical progress and diagnostic results. Within the “Then Focus” phase, the toolkit outlines five essential actions for clinicians to consider: Cease, discontinuing antibiotics if there is no evidence of infection; Amend, modifying therapy to a narrower or broader spectrum based on clinical findings; Refer, consulting or referring to specialised services when necessary; Extend, continuing treatment with a documented review or specified stop date; and Switch, transitioning from intravenous to oral antibiotics when appropriate. The 2023 update further reinforces the need for mandatory reviews within 24–72 h to ensure appropriate prescribing and reduce unnecessary antibiotic use (UK Health Security Agency, 2023). These measures are designed to enhance patient outcomes, optimise antimicrobial use, and mitigate the growing threat of AMR.

The COVID-19 pandemic significantly disrupted global healthcare systems, leading to increased inappropriate antibiotic use and rising AMR rates. There remains an urgent need to understand AMS practices during crises that disrupt healthcare delivery, particularly how AMS programs adapted to challenges such as staff shortages, resource constraints, and altered clinical priorities. This understanding is crucial for enhancing emergency preparedness and ensuring healthcare systems sustain effective antibiotic prescribing during future emergencies in secondary care settings (Elshenawy et al., 2024a).

Therefore, understanding how both seasonal variations and global crises influence antibiotic prescribing is essential for ensuring the effectiveness of AMS initiatives and combating the growing threat of AMR. This insight will be vital for developing robust AMS frameworks capable of withstanding disruptions and ensuring optimal antibiotic use during emergencies.

This pilot study aimed to evaluate the impact of seasonal variations, particularly the rise in respiratory infections during winter, and the COVID-19 pandemic on antibiotic prescribing patterns and antimicrobial stewardship practices in a UK secondary care setting. By comparing data collected before and during the pandemic. By comparing AMS practices from 2019 as a baseline with those during the 2020 crisis, the study sought to understand how AMS efforts were maintained or disrupted. The ultimate goal is to provide practical solutions and strengthen AMS practices to address the ongoing threat of antimicrobial resistance.

## Materials and methods

### Study design and setting

This pilot study aimed to assess the combined impact of seasonal variations and the COVID-19 pandemic on AMS practices by comparing data collected before and during the pandemic. Baseline data from 2019 served as a pre-pandemic reference, with measurements taken during the first week of March, June, September, and December. The exact periods were analysed in 2020, coinciding with UK national lockdowns and the initial rollout of COVID-19 vaccines in December 2020 (Institute for UK Government, 2022). Utilising an interrupted time-series approach, the study accounted for seasonal variations in antibiotic prescribing. This retrospective medical records review was conducted from 1 August 2021 to 28 February 2023 at a single National Health Service (NHS) Foundation Trust in the East of England, which serves a population of approximately 700,000 across 742 beds. The study focused on adult patients aged 25 years and above, aiming to assess AMS implementation and antibiotic prescribing patterns in 2019 (pre-pandemic) and 2020 (during the pandemic). The evaluation included AMS strategies outlined in the “Then Focus” phase, which emphasise examine Antimicrobial review outcomes based on the CARES framework and clinical progress.

### Study population (inclusion/exclusion criteria)

A stratified sampling strategy was employed to ensure maximum diversity among the included medical records. The inclusion criteria comprised adult patients aged 25 years and older, pregnant women, and immunocompromised individuals admitted to the Trust in 2019 and 2020. Only those prescribed antibiotics for RTIs, including pneumonia, were included in the study. Patients who spent less than 48–72 h in the Accident & Emergency (A&E) department were not prescribed antibiotics, or paediatric patients were excluded. This approach ensured a diverse and representative sample for evaluating antimicrobial stewardship practices.

The public and patient involvement included submitting the study protocol to the Citizens Senate, which provided valuable feedback and suggestions. This study was registered with the International Standard Randomised Controlled Trial Number (ISRCTN 14825813) and with Octopus, the global primary research registry (ISRCTN, 2022; Elshenawy, 2023). Ethical approval was granted by the University of Hertfordshire Ethics Committee and the Health Research Authority (HRA). Public and patient involvement included submission of the study protocol to the Citizens Senate, which provided valuable feedback and recommendations.

### Data sources and variables

In this retrospective cross-sectional study, patients were selected using electronic health records (EHRs) based on ICD-10 codes indicative of respiratory tract infections (RTIs). This included a range of conditions, encompassing both specific and indeterminate diagnoses. Specific conditions included community-acquired pneumonia (CAP), infective exacerbation of chronic obstructive pulmonary disease (COPD), hospital-acquired pneumonia (HAP), and ventilator-associated pneumonia (VAP). In 2020, the selection criteria were expanded to incorporate cases of COVID-19 pneumonia. Additionally, indeterminate diagnoses such as upper respiratory tract infections (URTIs), lower respiratory tract infections (LRTIs), and unspecified pneumonia were categorised as “Unspecific” RTIs. The primary diagnosis of RTIs in these records was crucial in determining the initial or empirical antibiotic prescribed to patients.

Utilising Minitab Statistical Software Version 21.1.0, and based on Public Health England’s estimation that 20% of all antibiotics prescribed in the UK might be inappropriate, with a 10% margin of error and a 95% confidence interval, the required sample size was determined (Public Health England, 2018). Data were randomly selected using Excel’s RAND function, resulting in a total of 80 patient records (40 from 2019 and 40 from 2020). This approach streamlined the sampling process while ensuring a comprehensive representation of the patient population. The primary author (RAE) extracted data from the EHRs, strictly adhering to the established inclusion and exclusion criteria. The extracted data included demographic characteristics and antibiotic prescribing practices, evaluated using the antimicrobial stewardship “Start Smart, Then Focus” Toolkit, which served as the study’s gold standard (UK Health Security Agency, 2023).

To validate the data extraction tool, two independent authors each extracted data from 10% of the sample (four patient records) per year, totalling eight records. An agreement rate of 80% or higher was required to confirm the tool’s validity (Price, 2018). For reliability assessment, both authors independently extracted data from 10% (eight records), and inter-rater reliability was determined by the percentage agreement. Discrepancies were resolved through discussion.

### Data collection

Data were collected from the medical records of 80 patients within the Foundation Trust in accordance with the specified inclusion and exclusion guidelines. Data were gathered from eight time points, specifically the first week of each selected month. The four pre-pandemic time points included: (i) March 2019 (Spring); (ii) June 2019 (Summer); (iii) September 2019 (Autumn); and (iv) December 2019 (Winter). Additionally, four pandemic time points were selected: (i) March 2020 (Spring)—the first wave of COVID-19; (ii) June 2020 (Summer)—the first lockdown; (iii) September 2020 (Autumn)—the second wave of the pandemic; and (iv) December 2020 (Winter)—the vaccination rollout. This approach ensured that data collection was consistent and accounted for seasonal variations and key phases of the COVID-19 pandemic.

### Data extraction

The primary author developed the data extraction tool by reviewing relevant literature and the UKHSA Toolkit. The authors collaboratively discussed and agreed upon the elements to be included in the tool. To extract data from patients meeting the inclusion criteria, access to the Trust’s electronic health system was necessary. Prior to commencing data extraction, the primary author completed training modules for these systems and subsequently gained access. The AMS data extraction tool encompassed demographic information, primary diagnosis, SSTF criteria, and AMS practices. This tool was employed to gather the required information from patients’ medical records, with each extraction taking approximately 45 min. This structured approach ensured the accurate and efficient collection of data necessary for assessing antimicrobial stewardship practices.

### Statistical methods

Descriptive analyses were conducted to summarise the data. Categorical and binary variables—including sex, age, admission speciality, patient classification, and types of AMS interventions—were presented as numbers (*n*) and proportions (%). Continuous variables with non-normal distributions were summarised using mean and standard deviation (SD). AMS implementation was assessed using the AMS Toolkit and further evaluated using the Start Smart Then Focus toolkit (UK Health Security Agency, 2023). Decisions made following this review were utilised to determine the type of AMS intervention. All statistical analyses were performed using Microsoft Excel 2019 for Windows (Microsoft, 2019).

## Results

Table 1 summarises the demographic characteristics and admissions of 80 patients. The cohort included 39 males (49%) and 41 females (51%), with a mean age of 76 ± 14.8 years, ranging from 26 to 99 years. Most patients were admitted to General Medicine (39 patients) and Elderly Medicine (18), with smaller numbers in Surgery (7), Cardiology (3), Respiratory Medicine (3), Accident & Emergency (1), and Others (1). The majority were urgent admissions (76 patients), while 4 were ordinary and routine admissions.

**Table 1:** Demographic characteristics and admissions (*n* = 80).

Characteristics		Admissions (*n* = 80)
Sex	Male (%)	39 (49%)
	Female (%)	41 (51%)
Admission specialty	General medicine	39
	Elderly medicine	18
	Surgery	7
	Cardiology	3
	Respiratory medicine	3
	Accident & emergency	1
	Others’^a^	1
Patient classification^b^	Ordinary and routine admission	4
	Urgent admission	76

a The “other” consultant specialities include endocrinology, diabetic medicine, acute internal medicine, thoracic medicine, neurology, and rheumatology.

b Ordinary admissions are planned and elective, while urgent admissions require immediate hospitalisation, often through accident & emergency (A&E), for acute illness or emergencies.

**Figure 1:**
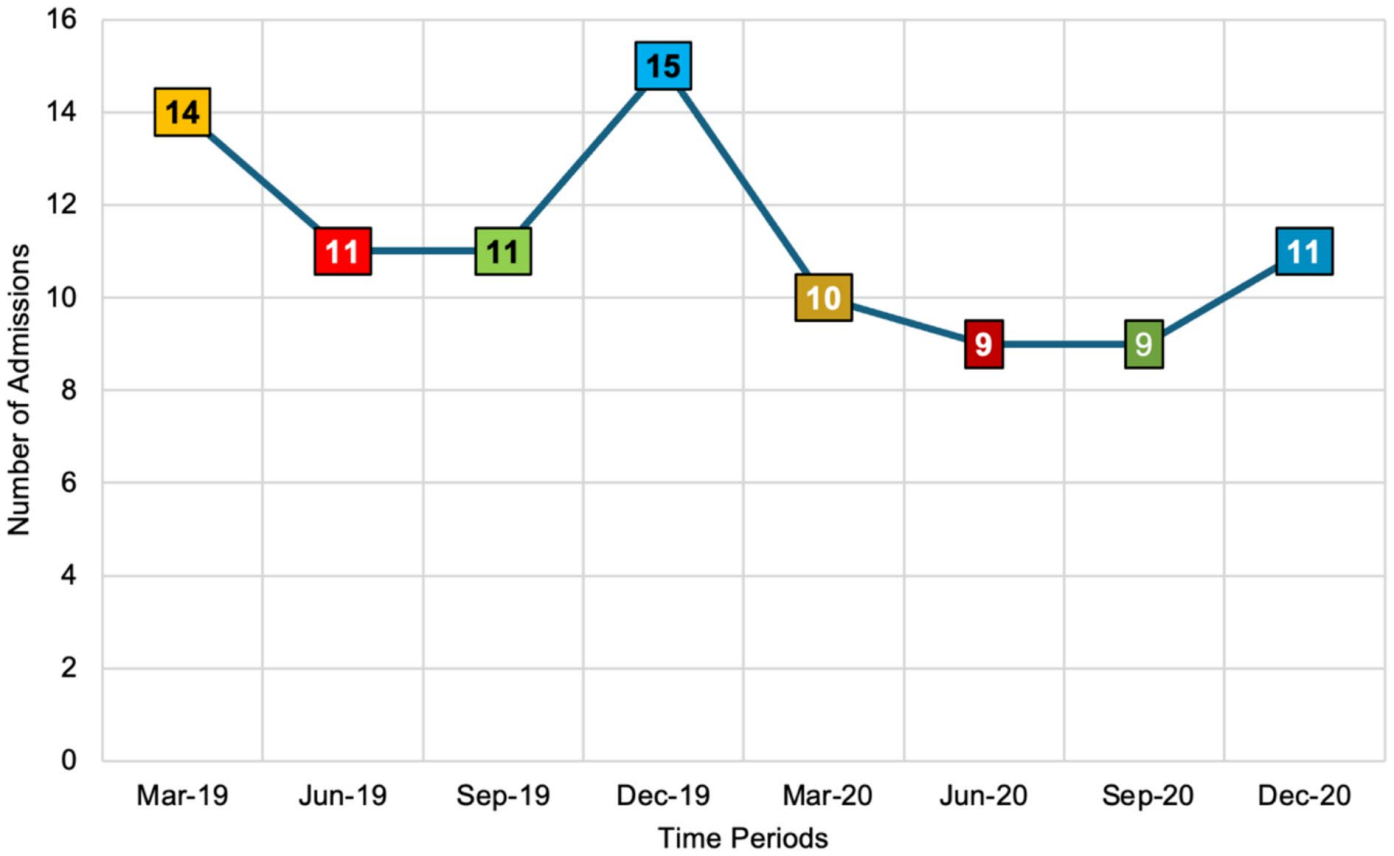
The number of respiratory tract infection admissions across eight seasonal time points in 2019 and 2020 (*n* = 80 admissions). This figure utilises a colour-coded scheme to illustrate the number of respiratory tract infection (RTI) admissions across different months and years. Orange, presented in both light and dark shades, represents RTI admissions in March 2019 and March 2020. Red, also in light and dark shades, denotes admissions in June 2019 and June 2020. Similarly, green, in light and dark shades, corresponds to RTI admissions in June 2019 and June 2020. Finally, blue, shown in both light and dark shades, signifies RTI admissions in December 2019 and December 2020. This colour differentiation allows for a clear visual comparison of RTI admission trends over the specified periods.

Figure 1 illustrates the number of respiratory tract infection (RTI) admissions during the first week of March, June, September, and December in both 2019 and 2020. Notably, admissions peaked in the first week of December 2019 with 15 cases. Although December 2019 marks the initial global emergence of the COVID-19 pandemic, the first confirmed COVID-19 case in the UK was reported in January 2020. Therefore, the December 2019 peak may not be directly attributable to COVID-19 within the UK context and could instead reflect typical seasonal variations or other factors influencing RTI admissions during that period. In the first week of March 2020, admissions decreased to 10, followed by a further decline to 9 in both June and September 2020. There was a slight increase in the first week of December 2020, with admissions rising to 11 cases. This pattern indicates fluctuations in RTI admissions that correlate more closely with the early stages and progression of the COVID-19 pandemic in the UK, particularly from January 2020 onward, rather than the initial global onset in December 2019.

Table 2 below compares the length of stay (LOS) in 2019 and 2020. The average LOS was almost the same between 2019 and 2020. The SD was 16 in 2019, while in 2020, the SD was 13.

**Table 2:** Length of stay in days (2019–2020).

Length of stay in days	2019	2020
Mean	16	15
Median	11	10
Range	1–119	1–97
Standards deviation	16	13

**Figure 2:**
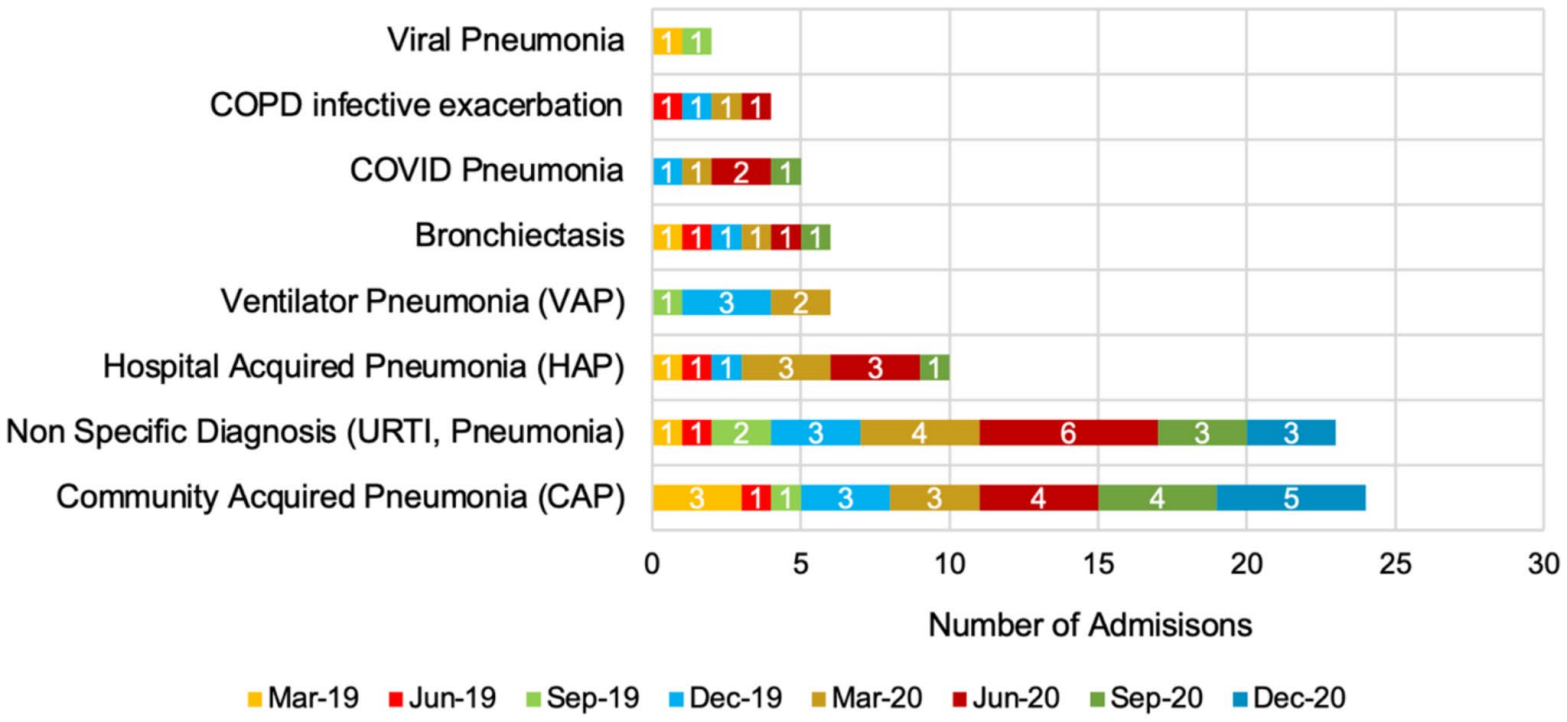
Seasonal trends in respiratory tract infection admissions in 2019 and 2020 (total number = 80 admissions).

Figure 2 presents the number of respiratory tract infection (RTI) admissions from March 2019 to December 2020, categorised by diagnosis and totalling 80 admissions. Community acquired pneumonia (CAP) was the most frequent, with 24 admissions, peaking at 5 in December 2020. Non-specific diagnoses (URTI, pneumonia) followed with 23 admissions, peaking at 6 in June 2020. Hospital acquired pneumonia (HAP) had 10 admissions, with peaks of 3 in both March and June 2020. Ventilator pneumonia (VAP) had six admissions, with 3 in June 2019. Bronchiectasis also had six admissions, evenly spread. COVID-19 pneumonia accounted for five admissions, peaking at 2 in March 2020. COPD infective exacerbation had four admissions, while viral pneumonia had the lowest frequency with two admissions, one each in March and September 2019.

**Figure 3:**
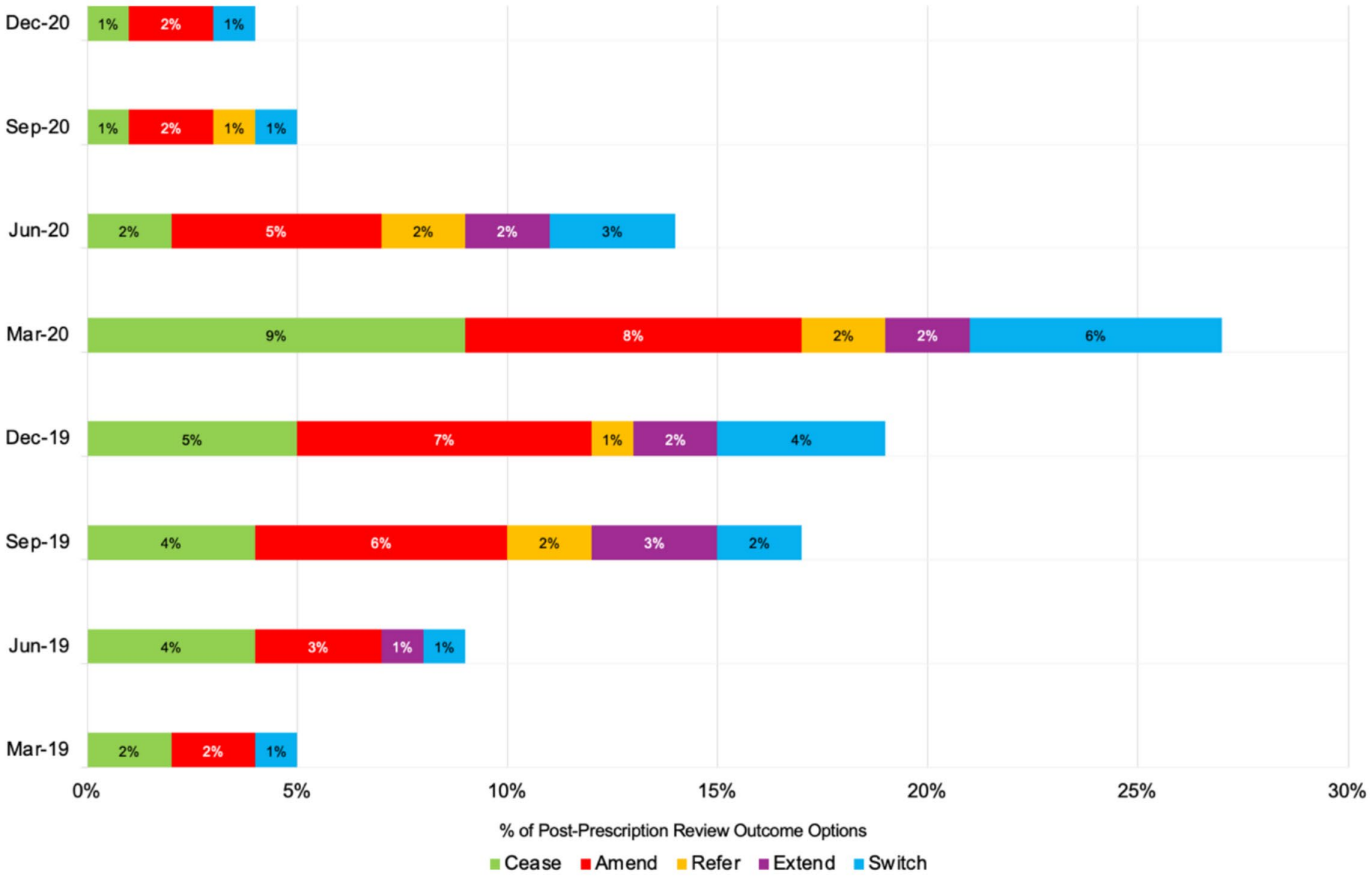
Number of post-prescription antibiotic review outcome options or AMS practices across eight seasonal time points in 2019 and 2020 (total number = 80 admissions).

Figure 3 illustrates the seasonal and monthly trends in antimicrobial stewardship (AMS) practices—Cease, Amend, Refer, Extend, and Switch—from spring 2019 (March) to winter 2020 (December). During the winter of 2019 (December), Cease actions were at 5%, peaking in early spring 2020 (March) at 9%, coinciding with the onset of the COVID-19 pandemic, before sharply declining to 1% by winter 2020 (December). A similar pattern was observed with Amend actions, which rose from 7% in winter 2019 (December) to 8% in early spring 2020 (March) and then declined to 2% by the end of winter 2020 (December). Throughout all seasons, the Refer and Extend actions remained consistently low, fluctuating between 1 and 3%. The Switch category, which indicates efforts to transition patients from intravenous to oral antibiotics, reached its peak of 6% in early spring 2020 (March) before decreasing to 1% by the winter of 2020 (December).

## Discussion

The findings from this pilot study provide valuable insights into the impact of the COVID-19 pandemic on antimicrobial stewardship practices in a secondary care setting in the UK. This study utilised an interrupted time-series analysis to compare antibiotic prescribing patterns before (2019) and during (2020) the pandemic, focusing on respiratory tract infections.

With regards to the demographic characteristics and admissions, this study included 80 patient admissions, with a balanced gender distribution (49% male, 51% female) and a mean age of 76 years. The majority of admissions were for general medicine (39) and elderly medicine (18), reflecting the high vulnerability of these groups to RTIs and the critical need for effective AMS practices in these areas. The pre-dominance of urgent admissions (76 out of 80) highlights the acute nature of these cases and the necessity for prompt and appropriate antibiotic use. A meta-analysis of 59 studies from the Netherlands in 2020, involving 36,470 patients, found that men and individuals aged 70 and above face higher risks of COVID-19 infection, severe disease, ICU admission, and death. The study highlights significant age and sex disparities in COVID-19 outcomes (Pijls et al., 2021).

Findings from this study illustrate the fluctuations in RTI admissions across eight time points in 2019 and 2020. The data shows a peak in admissions in December 2019, followed by a decline throughout 2020. While December 2019 marks the initial global emergence of COVID-19, the first confirmed case in the UK was reported in January 2020 (GOV.UK, 2022). Therefore, the peak in December 2019 may not be directly attributable to COVID-19 within the UK context but could instead reflect typical seasonal variations or other factors influencing RTI admissions during that period. As COVID-19 cases surged in the UK from early 2020 onward, several factors likely influenced the decline in RTI admissions. Increased awareness and testing for respiratory symptoms, changes in patient behaviour due to lockdowns, and restricted access to healthcare services may have contributed to this downward trend (Institute for UK Government, 2022). This pattern is consistent with findings from a 2020 study in the United States, which revealed a 33.7% decrease in daily hospital admissions for urgent conditions during the COVID-19 pandemic compared to 2019. Significant declines were observed in gastroenterology (−29.6%) and cardiovascular (−44.7%) admissions. These trends emphasise the critical importance of public awareness campaigns aimed at reassuring the public about the safety of seeking necessary medical care during pandemics. Ensuring that individuals feel safe to access healthcare services is essential to prevent declines in admissions for non-COVID-19 related urgent conditions, thereby maintaining overall healthcare system effectiveness and patient safety (Oseran et al., 2020).

For the LOS, the average LOS remained consistent between 2019 and 2020, with a mean of 15–16 days. However, the standard deviation decreased from 16 in 2019 to 13 in 2020, indicating a slight reduction in the variability of hospital stays during the pandemic. This could reflect more standardised treatment protocols or the impact of pandemic-related healthcare policies on patient management. A 2022 study in China examined 563,680 emergency admissions in 2020 and 709,583 in 2019, finding that the COVID-19 pandemic increased 28-day in-hospital mortality from 2.9 to 3.6%. The first and third waves had significantly higher mortality than inter-wave periods. The average length of stay decreased by 0.40 days, notably shorter for patients with mental disorders and cerebrovascular disease (Xiong et al., 2021).

The Centers for Disease Control and Prevention (CDC) report that during the COVID-19 pandemic, antibiotic prescribing in hospitals surged, with nearly 80% of COVID-19 admissions receiving antibiotics despite low rates of bacterial co-infections. While antibiotics and antifungals are essential for saving lives, their inappropriate use significantly contributes to increasing antimicrobial resistance (Centers for Disease Control and Prevention, 2021). For example, antibiotic misuse has been linked to the global rise of multidrug-resistant *Pseudomonas aeruginosa* (MDRPA) (Hayati et al., 2023).

Although overall antibiotic use decreased by August 2021 compared to 2019, prescriptions for specific antibiotics, such as azithromycin and ceftriaxone, increased, often being prescribed together. This trend likely reflects challenges in distinguishing COVID-19 from community-acquired pneumonia upon admission (Elshenawy et al., 2023a,b). Importantly, effective AMS strategies were implemented during the pandemic, aiding in maintaining appropriate antibiotic use and mitigating AMR risks. Sustained robust AMS practices are essential to ensure appropriate antibiotic prescribing and combat AMR in ongoing and future health crises (Elshenawy et al., 2024b).

For RTI diagnoses and antibiotic use, CAP was the most common diagnosis across the study period, with significant cases of HAP and non-specific RTIs. The emergence of COVID-19 pneumonia cases in 2020 highlights the direct impact of the pandemic on respiratory infection trends. The variability in diagnoses emphasises the challenges of maintaining precise AMS during a health crisis, emphasising the need for robust diagnostic and treatment protocols. As an example of pneumonia education, a Continuing Education Activity in Australia in 2024 highlights the complexities of bacterial pneumonia, including its symptoms, complications, and long-term impacts (Sattar et al., 2024). This module highlights a multidisciplinary approach to managing the disease, offering practical strategies for diagnosis, treatment, and patient care. It aims to enhance clinician knowledge, improve patient outcomes, and promote a cohesive healthcare approach.

Additionally, the protocol preparation adheres to national and international guidelines, including NICE guidelines, and incorporates results from local antibiograms (Sattar et al., 2024; NICE Guidelines, 2019). It is frequently updated with changes in local or national resistance patterns, clinical situations, or emergencies such as the COVID-19 pandemic. Updated protocols and antimicrobial guidelines should be properly disseminated to healthcare professionals to maintain proper antibiotic prescribing and antimicrobial stewardship practices (National Institute for Health and Care Excellence, 2023).

The COVID-19 pandemic posed significant challenges to maintaining optimal antibiotic stewardship. The consistent use of documentation for clinical indications and drug allergies is commendable, but the variability in other AMS interventions points to the need for strengthened protocols and continuous monitoring. These findings align with the study conducted in Spain in 2021, which reported increased inappropriate antibiotic use during the COVID-19 pandemic, highlighting a significant rise in inappropriate prescriptions and exacerbating antimicrobial resistance concerns (Calderón-Parra et al., 2021).

The seasonal and monthly trends observed in AMS practices reveal notable variations, particularly in response to the COVID-19 pandemic. Figure 3 shows that Cease actions, which indicate a complete cessation of antibiotic use, peaked at 9% in March 2020 and declined to 1% by December 2020. This increase in March aligns with the pandemic’s onset, reflecting heightened caution and AMS efforts during the crisis. Similarly, Amend actions peaked in March 2020 before tapering off, indicating that initial adjustments to antimicrobial treatments were made in response to the pandemic. The trends observed in this study align with findings from other studies. For example, the research on respiratory tract diagnoses in the United States found that antibiotic prescribing increased significantly during winter, driven by diagnoses where antibiotics were only sometimes or rarely indicated (Serletti et al., 2023). This pattern mirrors the peaks in AMS actions observed in the present study during the winter months of December 2019 and early spring 2020, which coincided with the pandemic onset.

Furthermore, the consistency of low Refer and Extend actions across all seasons, as seen in this study, suggests minimal referrals or extensions of treatment during the study period. A similar pattern of minimal seasonal variations in certain AMS actions was reported in the Netherlands’ study, which found that antimicrobial resistance rates in *Streptococcus pneumoniae* were higher in winter due to increased antibiotic use, similar to the peaks observed in this study (Martinez et al., 2019). The increased Switch actions in early spring 2020 indicate efforts to transition patients from intravenous to oral antibiotics, aligning with AMS goals to reduce inpatient antibiotic use. However, the subsequent decline in Switch actions reflects a stabilisation of AMS efforts as healthcare providers adapted to the ongoing pandemic challenges. Seasonal peaks of antimicrobial-resistant pathogens, such as vancomycin-resistant *enterococci* (VRE) and methicillin-resistant *Staphylococcus aureus* (MRSA) peaking in spring and *Klebsiella pneumoniae* and ciprofloxacin-resistant *E. coli* in summer, suggest the need for season-specific AMS strategies (Cassone et al., 2021). These findings emphasise the importance of understanding seasonal AMS trends to enhance stewardship strategies. The findings from this pilot study revealed that AMS practices were intensified during the pandemic and exhibited seasonal variations. This highlights the importance of implementing targeted interventions to address inappropriate prescribing and enhance AMS efforts consistently across healthcare settings.

This pilot study also acknowledges that incorporating detailed virological and microbiological data would enhance the interpretation of AMS interventions. In this pilot, only 5 out of 80 (6%) admissions were documented as COVID-19 pneumonia, while many records contained incomplete virology or microbiology data, limiting the ability to establish direct correlations between specific pathogens and prescribing interventions. This constraint highlights the need for future research to integrate comprehensive PCR and microbiological testing to provide a more detailed understanding of AMS practices in response to pathogen-specific infections. However, the primary aim of this study was to assess the feasibility of the data extraction tool and evaluate broader AMS patterns, rather than conduct an in-depth microbiological analysis. These findings highlight the necessity of future large-scale studies with improved microbiological reporting to refine AMS strategies further and ensure a more targeted approach to antibiotic prescribing, particularly during global health crises such as the COVID-19 pandemic.

### Strengths and limitations

This study has several strengths and limitations that influence its findings. One key strength is the role of pilot testing, which ensured feasibility, validity, and reliability in the study’s design and execution. The research highlights the impact of the COVID-19 pandemic on AMS practices, particularly in relation to seasonal variations, providing valuable insights to promote resilient and sustainable AMS frameworks, support rational antibiotic use, and address the global threat of antimicrobial resistance. The use of a validated data extraction tool and interrupted time-series analysis added rigour to the understanding of changes in AMS practices during the pandemic.

The study’s small sample size and single-centre scope limit generalisability. However, pilot studies play a critical role in testing feasibility and refining data collection tools before larger investigations. This study was designed as a pilot project to assess the reliability of a data extraction tool and evaluate trends in AMS practices. The manuscript explicitly identifies this as a pilot study to ensure clarity regarding its scope. Additionally, subsequent research, referenced in the Discussion, has since expanded the sample size and coverage, further addressing the feasibility findings of this pilot. Such pilot data are valuable for guiding resource allocation and justifying multi-centre or multi-year expansions. Pilot studies help optimise research methodologies, ensuring that future large-scale investigations are methodologically sound and effectively address antimicrobial stewardship challenges.

Despite these limitations, the study provides important insights into seasonal AMS variations during a global health crisis, demonstrating the necessity of tailored AMS strategies that adapt to seasonal and pandemic-driven disruptions in antibiotic prescribing. Future research should incorporate larger, multi-centre, and multi-year studies while also considering factors such as healthcare provider workload and hospital capacity, which may influence AMS adherence.

## Conclusion

This pilot study highlights the significant impact of the COVID-19 pandemic on AMS practices in a UK secondary care setting, particularly in relation to seasonal variations. Using interrupted time-series analysis, it examined antibiotic prescribing patterns for respiratory infections during the pre-pandemic period in 2019 and the pandemic period in 2020. Among the 80 admissions reviewed, community-acquired pneumonia was the most frequent diagnosis, with admissions peaking at 15 cases in December 2019, reflecting the seasonal burden of RTIs. During the pandemic, AMS interventions demonstrated notable shifts: Cease actions increased from winter 2019 to early spring 2020, Amend actions briefly spiked in March 2020, and Switch actions peaked in spring 2020. These findings highlight the need for targeted and adaptable AMS strategies to address seasonal trends and global health crises. Aligning AMS efforts with seasonal patterns and establishing robust, sustainable AMS frameworks are essential to saving lives and maintaining effective healthcare during global emergencies. Furthermore, the study emphasises the importance of pilot testing in ensuring research feasibility and reliability, ultimately advocating for sustainable AMS frameworks to combat antimicrobial resistance.

## Data Availability

The datasets presented in this article are not readily available because this data is restricted and confidential with the institution policy. Requests to access the datasets should be directed to: r.a.elshenawy@herts.ac.uk.
